# Enhancing Mental Health, Well-Being and Active Lifestyles of University Students by Means of Physical Activity and Exercise Research Programs

**DOI:** 10.3389/fpubh.2022.849093

**Published:** 2022-04-25

**Authors:** Cornelia Herbert

**Affiliations:** Applied Emotion and Motivation Psychology, Institute of Psychology and Education, Ulm University, Ulm, Germany

**Keywords:** physical activity, mental health, depression, well-being, low intensity exercise, perceived stress, emerging adults, university students

## Abstract

Mental disorders (e.g., depression) and sedentary behavior are increasing, also among emerging adults. One particular target group of emerging adults with high sitting times and vulnerability to mental disorders are university students. In particular, anxiety and depressive symptoms as well as stress symptoms are very common among university students. The present manuscript discusses whether physical activity and exercise interventions can help to promote the mental health of emerging adults such as university students. The manuscript will summarize current scientific evidence and based on this evidence, introduce an university-based scientific research project that investigates if physical activity, exercise interventions and acute bouts of exercise of low- to moderate intensity can buffer perceived stress, alleviate mental health symptoms and strengthen well-being (psychologically and physiologically) among university students by positively influencing depressive and anxiety symptoms, perceived stress and emotion perception, body awareness and subjective well-being including overall quality of life. The research project, its concept, multimethod approach, and first results from available studies are discussed in relation to current scientific evidence, health care needs and future developments. The results from the studies conducted within the research project so far and that are briefly summarized in this manuscript suggest that physical activity, mental health and well-being are positively related, also in university students as an important group of emerging adults. The results further suggest that exercise interventions comprising aerobic exercises of low- to moderate intensity may work best to improve mental health (alleviate depressive symptoms and perceived stress) among university students after a few weeks of intervention. In addition, acute bouts of certain types of exercises (yoga in particular) seem to be particularly effective in changing perception of bodily signals, cardiac activity and emotion processing immediately after the exercise. The results underscore the importance of systematic investigations of the combined examination of psychological and physiological factors that promote an active lifestyle and that strengthen mental health and well-being (psychologically and physiologically) among emerging adults such as university students.

## Introduction

Mental disorders such as depression (major depressive disorder, MDD) and non-communicable diseases (NCDs), most of them lifestyle related diseases (LSRD), are becoming the major causes of ill health (1) According to the World Health Organization (WHO), the prevalence of mental disorders has increased dramatically in recent decades, even in non-industrialized countries (1). Today, risk of mental disorders, depression (MDD) in particular, is no longer restricted to certain vulnerable population groups. Mental disorders such as depression (MDD) constitute a public health burden and a major cause of premature mortality and disability among all age groups and cultures [e.g., (2, 3)]. In Europe alone, the prevalence of depressive disorders ranges between 5 and 10%, respectively (4).

Negative changes in lifestyle and health behavior, emotional burden (stress), and reduced well-being including changes in affect, along with social, genetic, and demographic factors, are common factors involved in the onset and in the chronicity of many mental disorders such as MDD (5). As psychological and lifestyle related factors, many of these factors are not disease specific. They are significantly contributing to physical and mental health problems in general and impair physical, mental, and social performance, and quality of life of each individual. Therefore, mental health promotion is as important as is physical health promotion. There is no health without mental health (6).

Physical activity and regular exercise are essential for a positive, active and health-promoting lifestyle. According to the WHO, interventions aimed at increasing physical activity are sustainable health promoting interventions (7). Positive effects of physical activity and of regular exercise on health have been reported across all age groups in epidemiological studies and prospective, longitudinal follow-up studies [e.g., for an overview, (8, 9)]. As summarized in these studies, physical activity and the person's physical fitness are factors, both associated with a number of health improvements. Improvements can concern (a) physical health such as improved body composition, healthier lipoprotein profiles and cholesterol levels, better glucose, insulin and inflammation status, lower blood pressure, better cardiac functions and autonomic balance of the autonomous nervous system (ANS) and (b) improved and stable well-being (10, 11).

**Figure 1:**
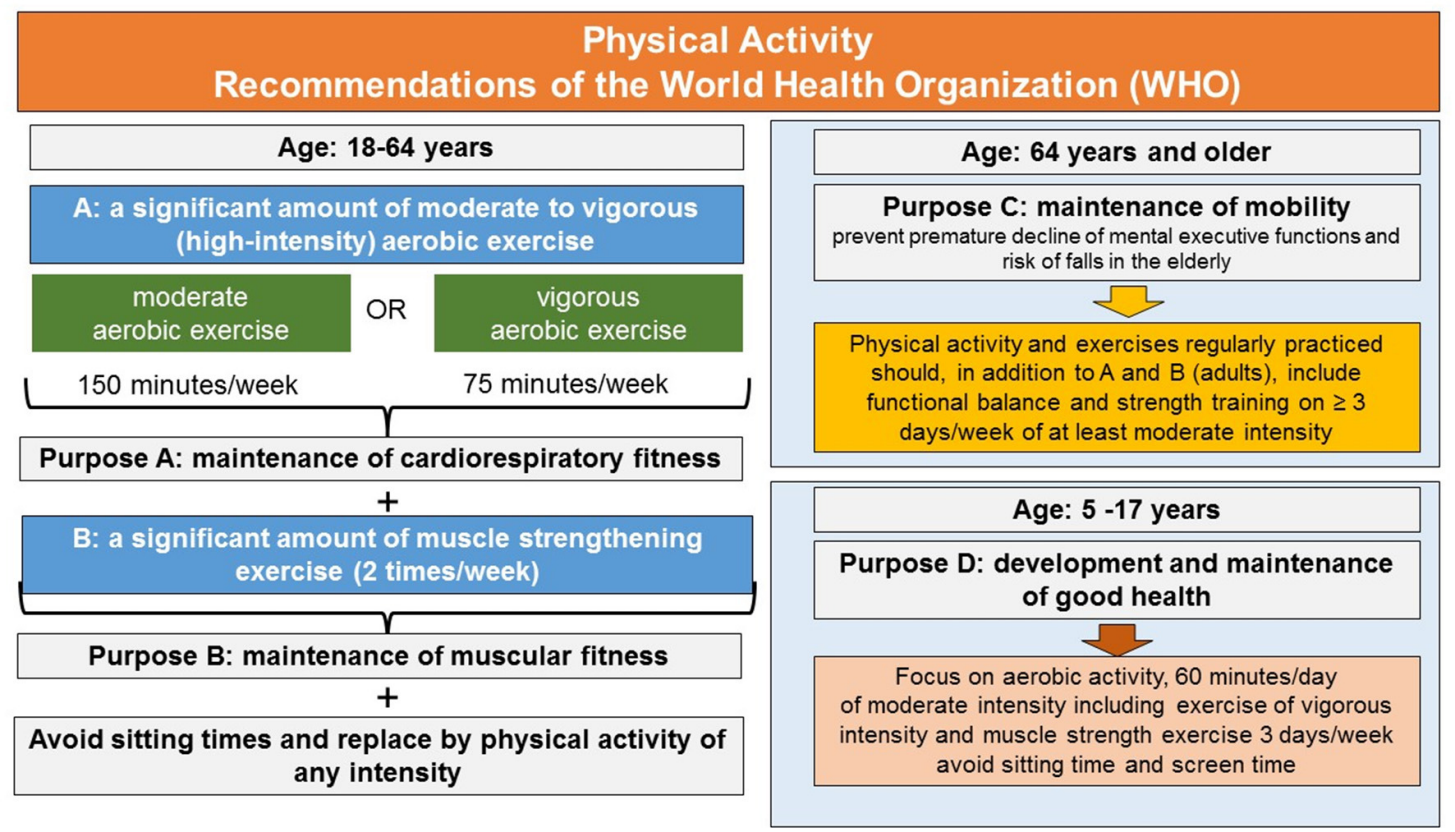
Physical activity and exercise recommendations of the World Health Organization. For details see text.

Based, among others, on this evidence, the WHO [latest version, see (7)] developed recommendations and guidelines for physical activity and regular exercise to provide the population and political, social and health-care decision-makers with general recommendations for health promotion. The physical activity guidelines distinguish between age and the degree or amount of physical activity and regular exercise required for health prevention, and the degree or amount of physical activity and regular exercise necessary to achieve health gain and health benefits beyond the basic health prevention level. As summarized in Figure 1, for health prevention, the physical activity and exercise should include (a) a significant amount of moderate to vigorous (high-intensity) aerobic exercise (e.g., endurance activities such as walking, swimming, treadmill running, or cycling) to improve cardiorespiratory fitness and (b) additionally include activities that foster muscle strength to improve muscular fitness. Furthermore, as people age, physical activity and the exercises regularly practiced should trigger somatosensory processing and neurocognitive functions as well. This is recommended to prevent the risk of falls and premature decline of mental executive functions in the elderly: executive functions are significantly involved in the cognitive control of walking and the maintenance of mental and motor mobility (12). In addition, the latest guidelines also make specific recommendations for vulnerable target groups and make recommendations how to increase physical activity of the population (7).

These basics rules are the major building blocks in almost any national physical activity guidelines and are also recommended by the American College of Sports Medicine (13). They are therefore in many countries current state of the art of health promotion initiatives and recommended by health care providers for daily practice to guarantee the maintenance of health and well-being in the general population.

The need for global physical activity recommendations for everyday life becomes reasonable when looking at the results of recent studies [e.g., (14, 15)]. These studies attest physical inactivity in a high proportion of the (world) population already at young age and during adulthood. Recent national health reports, for example for Germany, observed that about only 42.6% of the surveyed women and 48.0% of the surveyed men reported to meet the WHO's physical activity guidelines. In other words, they do reach at least 2.5 h of endurance activity per week during leisure time [e.g., (16)]. When the WHO's physical activity guidelines of weekly endurance and strength training are considered together, only few women and men in Germany and Europe women and men, aged between 18 and 84 years achieve the WHO's physical activity and exercise recommendation (17). When only considering adults at working age (18–64 years), even less women and men are able to exercise as recommended to maintain and promote a healthy and active lifestyle (17). Thus, physical activity recommendations comprising moderate intensity exercises are often not reached neither globally nor nationally nor individually and adherence to and maintenance of exercise recommendations is a general problem, well known in the literature [e.g., (18)], not only in the elderly.

In parallel with this global decrease in physical activity, daily periods of predominantly sedentary behavior, whether at work or during leisure time, have continuously increased across age groups and significantly in the majority of the young population [e.g., (19, 20)]. Significant relationships between a preferentially sedentary lifestyle of 4–8 h sitting time daily and negative mental and physical health outcomes have been confirmed by several studies for a number of health indicators (metabolic, cardiovascular, mental/psychological) (21). Further research suggests that a lifestyle characterized by excessive sitting, even in the presence of physical activity, contributes to an increased risk of chronic physical and mental conditions such as depression (22). The majority of the world's population already leads a physically inactive lifestyle (23). In a recent representative survey conducted among the adult population in Germany, on average, adults have been found to sit about 8 h per day [e.g., (24)]. There is consensus that a lifestyle characterized by sitting should be avoided at all costs (7). In line with this, experts including the WHO [see (7)] suggest that any physical activity could make an important contribution to the global endeavor of fighting sedentarism, and at the same time help to promote health and avoid illness among the population and age groups including emerging adults (25).

One particular target group of emerging adults at risk of mental disorders (26–28) and potentially also of increasing sedentarism (29–31) are university students. University students are young adults who, after completing their first educational career, pursue an academic education at a state or private university usually finishing with a bachelor's, master's degree or state examination. The aim is qualifying for an academic profession or pursuing a subsequent qualification for an academic degree. The average years spent at university is 6 years. The workload during this time is high. The accumulated work load for instance in a bachelor's degree corresponds to 45–56.25 h/week, a time spent predominantly in terms of sedentary activities in lecture halls, seminar rooms, or at the desk at home. On average, this results in a daily sitting time of approximately 6.4–11.25 h, 5-days a week. This sitting time has been approved in international studies (29–32). Recent international surveys have reported an increase in mental health complaints and perceived stress among university students. According to resent survey estimates, worldwide about every fifth student reports anxiety and depressive symptoms and just as many students report to seek help for coping with academic stress and mental health conditions [e.g., (27, 28)]. Statistics from university counseling centers complement these numbers showing that roughly over half of the counseling cases of the clients (university students) report already seeking therapeutic help. However, according to recent surveys, most mental disorders among college students aged 18–22 years are untreated (26). The current Covid-19 pandemic is expected to increase these numbers in emerging adults (33) and among university students (34). Worldwide, 2.7% of the total world population are university students. In future, an increase of 30 million of university students per year is expected globally due to improved access to education. Accordingly, university students as emerging adults with high cognitive demands, high self-reported psychological stress, and high weekly sedentary time may constitute an at-risk group in primary health care prevention who could benefit from exercise interventions in the short as well as in long run.

However, which type of physical activity or exercise might be most effective for mental health promotion during adulthood, in particular in emerging adults such as university students?

The positive health effects of regular exercise of moderate to high and vigorous intensity exercise that builds on aerobic endurance and muscle strength exercise in an amount and daily/weekly regularity recommended by the WHO guidelines (see Figure 1) have been as outlined above investigated intensively in the past in scientific research. Undisputed are the results from epidemiologic studies according to which physically active people who report practicing a daily or weekly routine of regular exercise of moderate to high intensity have permanently reduced impairments in general health and reduced mortality risk [e.g., see for an overview (35, 36)].

In line with these observations, recent review- and meta-analytic studies have revealed promising effects of moderate intensity exercise for alleviating depressive symptoms in the treatment of patients with clinical diagnosis of depression (e.g., major depressive disorders, MDD) [e.g., for an overview (37, 38)]. These positive effects associated with regular exercise of moderate intensity encouraged medical recommendations of exercise in the treatment of mental disorders such as MDD in addition to or in conjunct with treatment as usual (psychotherapy and pharmacological treatment). The prescriptions of exercise interventions have been included already in national guidelines. One prominent example is the UK National Institute of Health and Clinical Excellence (39), who included exercise interventions in their report for the treatment and management of depression in adults. The report reviews the current clinical evidence and based on it attempts to formulate exercise recommendations for the treatment of depressive symptoms (39). The recommendations address patients with a clinical diagnosis–irrespective of age-and with persistent mild to moderate depressive symptoms who should exercise 2–3 times a week for at least 45–60 min per session for at least 10–14 weeks to achieve improvement in the severity of depressive symptoms. The exercise should in line with the WHO's criteria (Figure 1) involve endurance and resistance exercise. A health care professional should supervise the exercise because in most studies, supervised-exercise interventions have proven superior over unsupervised exercise interventions in these patient groups. Regarding treatment of anxiety disorders by means of exercise, current evidence is less consistent than that for the treatment of depression, but there is evidence that state anxiety is significantly reduced after acute bouts of moderate intensity exercise [e.g., (40)].

In summary, the recommendations of moderate intensity exercise for mental health promotion are strengthened by findings that exercise of moderate intensity has comparable effects in lowering symptoms as pharmacological treatment with e.g., antidepressants in patients with MDD has (41, 42). Therefore, a common opinion and hypothesis is that dose-response relationships significantly matter, not only for achieving benefits in physical health but in mental health as well. In other words, it is assumed that if the exercises or the physical activity carried out during an exercise session or exercise intervention have no metabolic and no physiological effects, they cannot have any direct effect on physical health or on mental health for achieving changes in the physical symptoms, the physiological symptoms or the neurobiology of mental disorders. In fact, mental disorders such as major depressive disorder (MDD) or anxiety are not just mental disorders. As affective disorders, the key symptoms of for example depression comprise depressed mood and loss of interest in nearly almost all activities approximately all days and these key symptoms affect the whole person and organism (DSM-5, Diagnostic and Statistical Manual of Mental Disorders, Fifth Edition). Mood changes and loss of interest occur in companion with changes in psychomotor and sleep patterns, appetite and concentration, suicidal ideation, feelings of worthlessness and low self-esteem (DSM-5). Moreover, these physical and mental changes are accompanied by changes in neurotransmitters, by changes in brain functions and by changes in activity in prefrontal brain regions and subcortical reward and memory brain systems, i.e., brain regions and neuronal networks controlling emotions, mood, motivation, cognition, behavior and affect. Moreover, depression-related changes in peripheral-physiological bodily functions such as changes in blood pressure and heart rate variability (HRV), changes in cortisol levels and inflammatory parameters speak to altered functioning of the central, the autonomous nervous system (ANS) and the immune system in MDD [e.g., (43)].

So far, however, there is only little systematic research about the efficacy of exercise interventions in the field of primary prevention of health among the adult age groups, who are yet not suffering from clinical symptoms [for overviews see for example (36, 44, 45)]. A recent publication (46) summarizing data from the HUNT cohort prospective follow-up studies is one of the few studies that investigated the relationship between exercise and mental health in healthy adults and controlled for the role of exercise related dose-response effects. The publication (46) included a sample of 33 908 healthy, never depressed adults, with a mean age of 45.2 years (SD = 16.5 years) who were investigated two times in a time window of 11 years. The results suggest that exercise of low intensity of about 1 h (walking) exercise a week to be effective in reducing the risk of depression by 12% with little or no significant additional depression alleviating benefits of exercise with a duration beyond 1 h a week. Similar promising effects that low-intensity exercise interventions comprising a diversity of exercise types (aerobic exercise, yoga, dancing, resistance training, etc.) can alleviate stress- and depressive symptoms and improve mental health across a wide age range, comes from recent reviews of meta-analytic studies (44, 47). The meta-analyses suggest that a diversity of different types of exercise, specifically, mind-body exercises such as yoga have proven equivalent to aerobic moderate intensity exercise such as walking, swimming or cycling, in improving mental health at least in clinical groups or vulnerable groups at risk of mental ill health.

As far as the treatment of mental disorders in emerging adults by means of exercise interventions is concerned, still few systematic evidence is available so far. A recent scoping review suggests that the current evidence is restricted to only a few studies (48). Moreover, concerning primary prevention of health in emerging adults, evidence that physical activity, mental health and well-being are related comes mainly from self-report studies [e.g., (49)]. In addition, systematic research evaluating the effectiveness of exercise interventions on mental health of university students, depression in particular, seems also still limited (50). A recent meta-analysis compared seven exercise interventions for their effectivity in alleviating depressive symptoms in undergraduate college students and found that mind-body exercises such as Tai Chi and yoga were best suited to reduce depressive symptoms compared to team sports such as basketball or badminton or dance (50) in the targeted groups.

## AIM and Scope of the Present Manuscript

As summarized in the previous section, the following important and significant question remains: can exercise interventions of low- to moderate intensity and short duration as well as acute bouts of exercise make an important contribution to the primary prevention and the promotion of mental health and well-being of emerging adults such as university students, yet not suffering from clinically relevant mental health conditions? In this manuscript, first results from a still ongoing psychological research project (Anem Fit&Well) will be summarized that provides first answers to this question. The research project (Anem Fit&Well) and its studies examine the relationships between physical activity, different types of acute and regular exercise, mental health and well-being in healthy adults with a specific focus on emerging adults including university students.

In the following sections, the research project, its concept, methods, and first results from available, already published studies are summarized and discussed in relation to current scientific evidence, health care needs and future developments. In particular, the results from 5 studies will be summarized that investigated if physical activity, exercise interventions and acute bouts of exercise of low- to moderate intensity can buffer perceived stress, alleviate self-reported symptoms of mental health conditions such as depressive and anxiety symptoms, change cardiovascular activity during exercise and at rest, and strengthen subjective well-being by positively influencing mood and affect, emotion perception, body awareness, and overall quality of life.

The first section under Materials and Methods will describe briefly the general framework of the research project in terms of the theoretical understanding of concepts, the methodological approach including systematic classification of the exercises chosen in the research project and its studies. Next, the study designs, the participant samples, inclusion and exclusion criteria of the already available studies are described and the major results and conclusion drawn from the results are summarized in the Results and Discussion. Finally, a future outlook will be provided and the aims of the research project will be discussed in relation to health care needs and future developments.

## Materials and Methods

### Conceptual Framework of the Project

**Figure 2:**
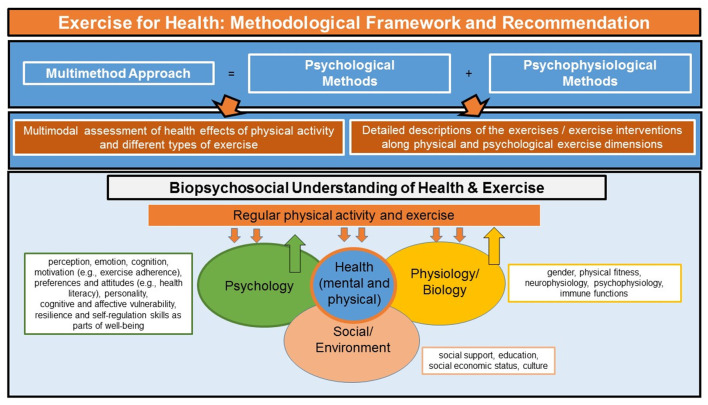
Exercise for health prevention. Overview of the theoretical and methodological approach of the research project. For details see text.

Theoretically, the research projects builds on a biopsychosocial model and understanding of health. A biopsychosocial understanding of the effects of exercise on mental health allows to take physical and mental processes and their interaction into consideration. As illustrated in Figure 2, this is critical for the understanding of how psychological and physiological mechanisms interact at different exercise intensities to promote mental health and well-being. So far, most studies and most research programs focus on either the psychological or the physiological mechanisms of exercise on mental health. Several physiological and psychological variables have been suggested to play a role [e.g., (51)]. Therefore, and as summarized in Figure 2, methodologically, the present research project and its scientific studies are following a multimethod approach that combines psychological methods and psychophysiological methods to investigate exercise-related health effects on behavior, physiology, and subjective experience in well controlled laboratory and field (survey, online) experiments.

### Exercise Classification and Methodology

There is a huge variation across exercise studies in the way physical activity and the individual exercises included in the exercise interventions are described. The exercise descriptions of the study protocols of previous studies often vary in terms of the outcome variables investigated in a particular study. They vary across the research disciplines that carry out the research (medicine, psychology, health and life science, etc.), and the exercise descriptions vary with the preconditions of measures available in a lab or research team/environment. This makes comparison across studies difficult. Therefore, one major attempt of the research project Anem Fit&Well is to describe the individual exercises and exercise interventions included in its studies as best as possible according to the major dimensions of exercise including type of exercise, exercise duration, and intensity (52, 53). Moreover, frequency, density and duration of the exercise sessions (its repetitions) are included if the exercise comprises more than one exercise session. Notably, all studies included in the research project follow the scientific nomenclature (52, 53): the term physical activity (PA) is used for the description of any activity that is carried out on a regular day including activities from or to work. In the following sections, the term physical activity (PA) is used whenever to describe the habitual physical activity levels of the participants engaged as study participants. The term “exercise” is used for the exercises included in the research project and its exercise intervention studies that are carried out as planned, structured, repetitive and intentional movements with the intention to explicitly improve or maintain mental health (52, 53). Like for each individual exercise of the research project, the exercise interventions are described along fundamental exercise dimensions including frequency of the individual exercise sessions, number of repetitions of a session per day or weeks (daily and weekly practice). The exercise protocols allow comparison with physical activity recommendations from the literature examining the relationship between mental health, physical activity and exercise.

### Standardized Assessment of Type of Exercise and Exercise Intensity

**Figure 3:**
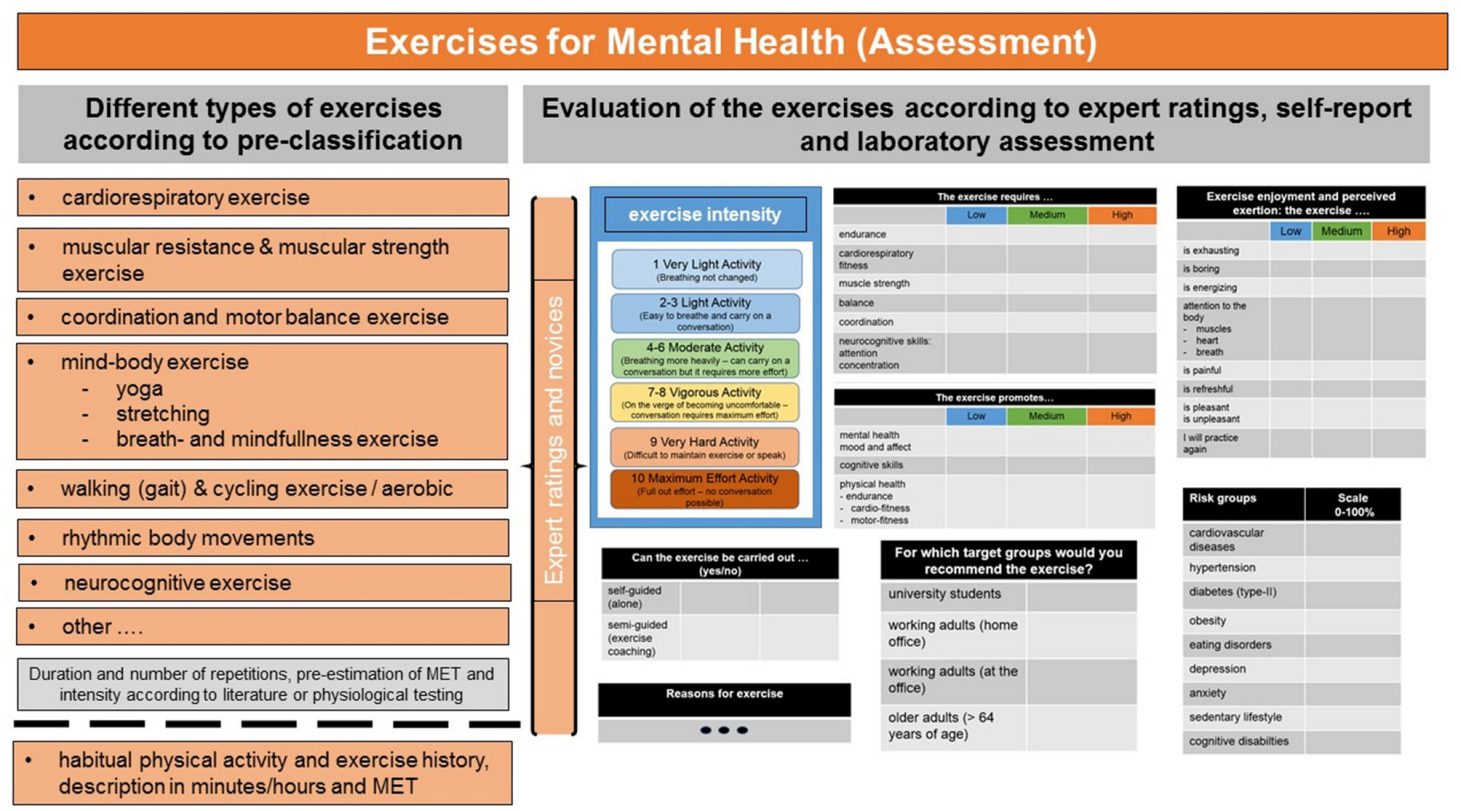
Different types of exercises included in the research project. Pre-classification and evaluation of the exercises according to exercise dimensions, expert ratings and laboratory assessment. For details see text.

The individual exercises included in the research project comprise different types of exercises [e.g., cardio, resistance, coordination and balance, ergometer or treadmill exercise, mind-body exercise (e.g., yoga)] whose movements (isometric, isotonic, or isokinetic) and activity carried out can load on the different exercise dimensions (cardiorespiratory, endurance, muscular strength, flexibility, speed, balance, and coordination) in varying degrees and whose intensity and duration range above rest (> 1.5 MET), but below, at or if whished, above the physiologically and metabolically effective stimulus thresholds (1.5–6 MET or beyond; short: MET, metabolic equivalents) see Figure 3 for examples^1^.

Objective measures of exercise intensity (e.g., measures of MET and of energy expenditure such as VO_2_ max, heart rate, respiration) might not be available in all research units or research labs, furthermore these measures are often difficult to obtain in online studies without wearable devices for monitoring and recording of these measures. This makes cross-comparison of exercises and exercises interventions described in the literature often difficult. To avoid this lack of standardization, in the current research project, exercise professionals and novices additionally rate the individual exercises on pre-chosen standardized exercise dimensions. Expert ratings are an excellent means and research tool for providing valid descriptions of the exercises from an expert's point of view (54). The Borg exercise scale(s) of perceived exertion (55) ask for bodily and cognitive exercise symptoms such as changes in heart rate, respiration or breathing rate, sweating, or (muscle) fatigue. The scales are often used as valid self-report methods to classify exercises. The Borg scale has received good validity and can be used by health care experts or by novices for exercise ratings. Its scores corresponds well with the objective measure of the degree of heart rate changes elicited by the exercise, at least for aerobic exercises (55). Its verbal descriptions allow categorization of exercise activities into low, moderate and high intensity. Moreover, besides the evaluation by experts and novices, the individual exercises are compared to exercise descriptions in the literature. For example, the general classification schemes of physical activity provided by the Compendium of physical activities (56) comprise a plethora of physical activities grouped into daily physical activities and sports activities (exercise planned and structured). The Compendium provides MET values for each activity, and based on these, the activities are classified as low, moderate or high in intensity. Of note, the classification system of the Compendium of physical activities can only provide rough average estimates of categorization of activities into those of low, moderate or high intensity. In the present research project, the classification of the exercises based on the compendium (56) is included as heuristic additional source of comparison.

### Standardized Assessment of Mental Health, Perceived Stress, Well-Being and Habitual Physical Activity

**Figure 4:**
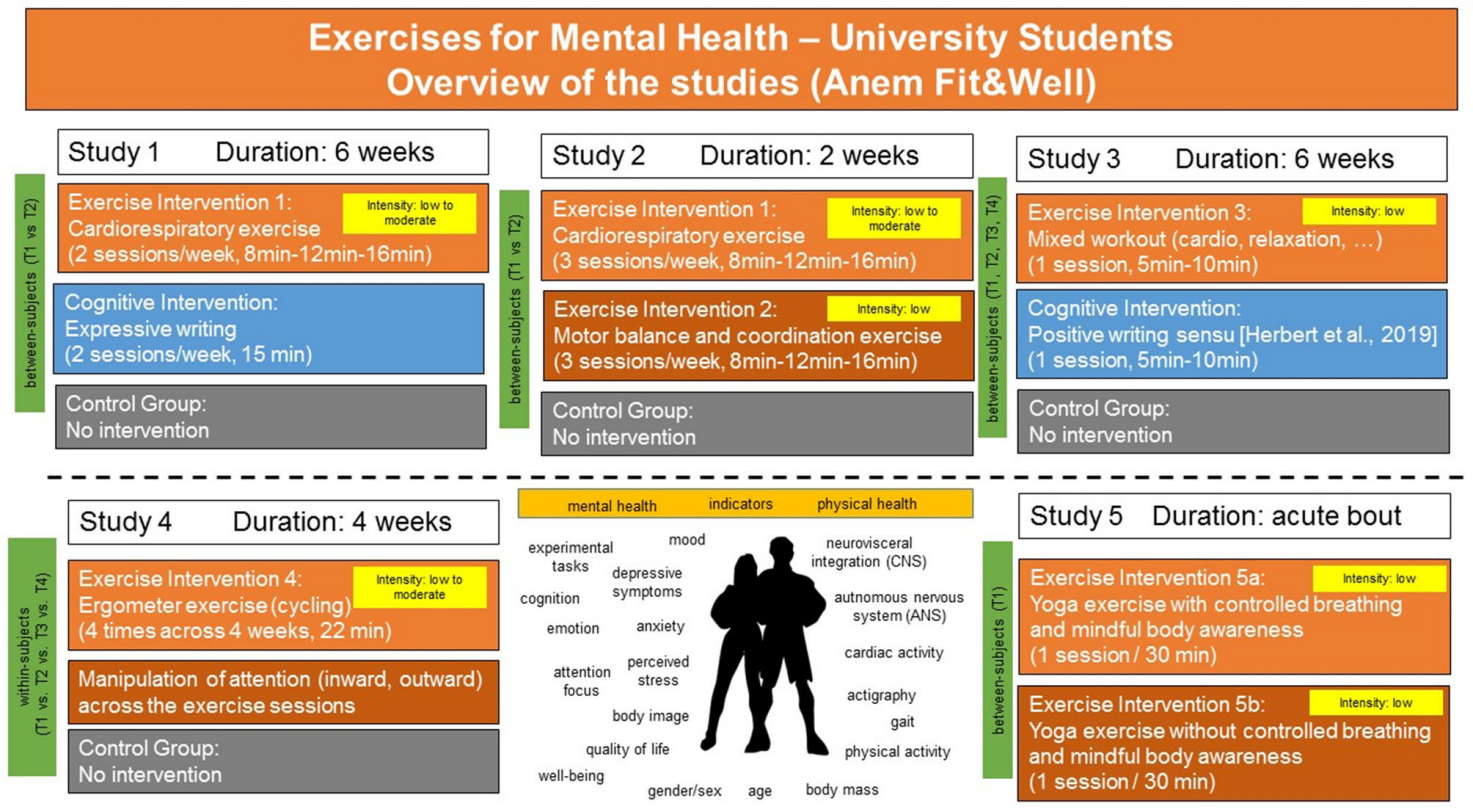
Overview of the intervention studies of the research project. For details see text.

Importantly within and across the scientific studies of the research project, a number of standardized psychological self-report measures as well psychological-experimental tasks and tests are included for profound mental health assessment and for capturing changes in perceived stress as well as in in other psychological domains including well-being and quality of life. Of note, mental health, well-being and quality of life are broad constructs, especially well-being and quality of life are often divided into subdomains of physical and psychological well-being or objective and subjective measures of quality of life, the latter often comprising aspects of physical and mental health and well-being (e.g., material and physical well-being) as well. In addition, subjective well-being focuses on several factors such as mood, affect or emotions as well as a person's capacity, resilience and satisfaction with one's life [e.g., (57)]. As briefly illustrated in Figure 4, the research project and its studies aim to assess mental health, stress, and several facets of quality of life and subjective well-being with standardized questionnaires in addition to using standardized questionnaires for detailed profiling of depressive symptoms, anxiety, mood, body awareness and body image to name but a few of the dimensions included in the project's psychological assessment. In addition, the psychological assessment is not only based on self-report measures but aims to include experimental psychological paradigms to capture immediate changes in perception, cognition, and emotion or body awareness, during or after the exercise intervention compared to pre-exercise. In contrast to self-report measures, experimental investigations include objective outcome variables such as reaction times or task accuracy that can be further combined with measuring changes in brain activity or heart rate for psychophysiological assessment. Inclusion of a battery of self-report measures and assessment tools that comprise standardized self-report questions and short experimental investigations can give comprehensive insight into exercise-driven mental health effects. Moreover, the default assessment in the project includes the assessment of the participants' habitual physical activity level. In addition, each participant is screened for his/her physical activity habits, and, if possible (e.g., in the laboratory), for cardiovascular/cardiorespiratory fitness according to standardized in house anamnestic screening protocols. From a biopsychological understanding of health undertaken in this research project, only a full assessment of this kind can improve understanding of the when and why physical activity and exercise interventions promote health and well-being in healthy adults.

### Study Description and Study Design

In summary, the study design of five intervention studies conducted within the research project and whose results have already been published separately, all investigating university students (gender-mixed samples or all-female samples) and some including randomized control trial studies (RCTs) are described in the following sections and their main findings are outlined in a brief review in the Results section. The original results of these studies can be found in the following publications (58–62). An overview of the studies and the study designs is illustrated in Figure 4. As illustrated in Figure 4, study 1, study 2 and study 3 were using similar study protocols comparing exercise interventions against cognitive interventions or against a non-exercise control group (59, 61). Study 1, study 2 and study 3 were carried out during the time course of a semester, i.e., when university students were at the campus and actively enrolled in their studies. Study 1 was carried out as an online intervention, study 2 was carried out in the laboratory (59) and study 3 (61) was carried out weekly once within the classroom across the term. Study 1 and study 2 were RCTs.

#### Study 1 Cardio Exercise vs. Cognitive Intervention vs. Wait List

In study 1, all interventions (exercise, cognitive intervention or wait list) lasted 6 weeks. In study 2 all interventions lasted 2 weeks. In study 1, the exercise intervention as well as the cognitive intervention comprised 12 sessions within 6 weeks. The 12 exercise sessions increased in duration across the 12 sessions from 8, 12, to 16 min, respectively. The cognitive intervention and wait list followed the same time schedule. The exercise intervention was semi-guided by a female and male exercise model at the age of the volunteers (all university students, women and men). The exercise sessions were delivered as exercise videos. The exercise intensity of the exercises included in study 1 were evaluated in a separate sample of volunteers by means of heart rate measures. Moreover, the exercises were evaluated by experts in a separate study (60). This suggested that the 16 min exercise sessions of study 1 (59) were at moderate intensity and suited to increase cardiorespiratory fitness and muscular strength.

#### Study 2 Cardio Exercise vs. Motor Balance and Coordination Exercise vs. Wait List

To better understand the contributions of the type of exercise and the contribution of the duration of the exercise intervention to mental health improvements, the exercises included in study 1 were also used in study 2 and in study 2 compared against an exercise intervention comprising balance, coordination and motor training. The effects of the exercise interventions in study 2 were compared against a wait list control group and in contrast to study 1, the exercise interventions were carried out across a period of 2 weeks only, with 3 sessions per week (see Figure 4). Thus, in study 2, the exercises of study 1 were carried out at the same intensities but the individual exercise sessions (frequency) was increased to 2 weeks instead of 6 weeks. Akin to the participants of study 1, the participants of study 2 performed the interventions on the same days, hours etc. to control and exclude as many confounding factors as possible (changes in circadian rhythm, etc). Moreover, study 2 included detailed psychophysiological assessment of parameters of cardiovascular fitness of the participants and improvements therein pre- to post intervention. Finally, participants of study 2 received the same psychological assessment battery of questionnaires as the participants of study 1, and although study 2 was conducted in the laboratory, the exercises were presented akin to study 1 as semi-guided exercise videos [for an overview see, (59)].

#### Study 3 Short Physical Activity Breaks vs. Cognitive Intervention

In study 3, the duration and frequency of the interventions were reduced to 1 session/week, the interventions were carried out weekly once within the classroom across the winter term (1 session per week, starting at T1, T2 until T3; the distance between T1, T2 and T3 was 3 weeks, follow-up at T4), guided by an exercise model. The exercise interventions were carried out at the beginning of the class and lasted about 5 min and no longer than 10 min. Moreover, in contrast to study 1 and study 2, the exercises included in study 3 consisted of a mix of exercises of low intensity that comprised muscle relaxation and other types of activities such as simple dance steps [see (61); and Figure 4 for an overview and summary].

The cognitive intervention included in study 1 and study 3 comprised an expressive writing task (12 sessions, 6 weeks, study 1 or 1 session weekly across the winter term, study 3). The participants in the expressive writing group of study 1 wrote about their most stressful weekly events (59). In study 3, the cognitive intervention included writing about positive events following the in-house protocol of the author of this manuscript. The effects of a single session of expressive writing about stressful events as well as of positive writing used in study 1 and study 3 is described in detail (63). The writing intervention of study 1 was carried out online, whereas in study 3, it was akin to the exercise session carried out in the classroom, weekly, starting at T1, T2 until T3 with a distance of 3 weeks between T1, T2, and T3, respectively. Like in study 1 and study 2, the participants of study 3 underwent detailed psychological assessment including assessment of global physical activity, depressive symptoms, and quality of life.

#### Study 4 Acute Bouts of Cycling Exercise of Moderate Intensity vs. Control Group

Whereas, study 1, study 2 and study 3 used individual exercises comprising aerobic cardio fitness exercises, motor balance and coordination training, and relaxation exercise (study 3 only) to investigate specifically the mental health benefits of different types of exercises combined in the interventions, the laboratory study 4 (62) investigated the effectiveness of 4 sessions of ergometer exercise (bicycling of moderate intensity) in a within study design in which the same participants performed all exercise sessions and the control condition.

#### Study 5 Mind-Body Exercise (Yoga) With Controlled and Mindful Breathing vs. Without

The laboratory study 5 (64) used yoga exercises with and without controlled breathing and mindfulness instructions as mind-body exercise intervention and investigated the effects of the exercise after a single exercise session (see Figure 4). Moreover, akin to study 2, the laboratory study 4 and the laboratory study 5 included detailed psychophysiological assessment of cardiovascular improvements pre- to post intervention and a set of experimental tasks allowing first answers to the question of the role psychophysiological interactions play in the improvement of cognitive-affective processing, and body awareness after single sessions of low to moderate intensity exercise.

#### Study Participants, Inclusion and Exclusion Criteria

Study 1 comprised a mixed gender sample, study 3 aimed at including women and men, and study 2, 4 and 5 comprised all female samples (to keep psychophysiological recordings constant). All participants were screened for mental and physical health conditions because a history of mental or physical disorders and past and current injuries or cardiovascular disorders and other health conditions were exclusion criteria. An age of at least 18 years was an inclusion criterion as was German as native language. Study 1 comprised *n* = 153 university students (127 women), study 2 comprised *n* = 32 university students (*n* = 2 men), study 3 comprised *n* = 105 university students, whereas study 4 included *n* = 30 university students (all-female) and study 4 included *n* = 34 university students (all-female sample). In study 1 and study 3, participants with missing data or drop outs were excluded from the final analysis of pre-post intervention effects, resulting in smaller sample sizes, especially in study 3 [for a detailed overview of the study design and drop outs see (59, 61)].

## Results

### Habitual Physical Activity Behavior and Sedentarism Among University Students

As summarized in this section and in Table 1, the RCT studies, i.e., study 1 (mixed-gender sample) and study 2 (all-female sample) yielded an overall sitting time of 7.45 h/day (study 1) and 7.6 h/day (study 2) in the samples of university students. Averaged across the study sample, 23.94% and 26.76% of the participants of study 1 reported to not engage in any vigorous or moderately intensive activities in their free time. Regarding the total duration of their weekly physical activity (i.e., including activities at work, during transport or leisure), 14.79% participants of study 1 and 30% of the all-female sample of study 2 did not reach the WHO guidelines of 150 min of moderate-intensive physical activity, and about 7% (study 1) reported to not engage in vigorous activity and this held true for both, women and men [for an overview see (59)]. Study 3 confirmed this trend in sedentary behavior (574.62 min/week vs. 638.57 min/week corresponding to 9.6–10.64 h/week sitting). Thirty-five percentage of the university students reported to spend between 15 and 25 h/week at the university and akin to the participants of the study 1 and study 2, the participants of the study 3 reported to be physically active only about 62.86–137.96 min/day and 15% in total did not reach the WHO recommendations. Seventy-four percentage of the all-female sample of study 5 (64) reported to spend at least once a week in regular exercise activities such as jogging, swimming, cycling, dancing, team sports, martial arts, strength training, balance, or gymnastics.

**Table 1:** Overview of the results of the studies (study 1 and study 2) of the research project assessing prevalence of physical activity **(A)**, self-reported depressive, anxiety and stress symptoms **(B)** and the relationship between habitual physical activity, mental health and well-being **(C)** among university students.

**A. Sedentarisms and physical activity**	
**Average sitting time (hours per day/week)**	**7.45–7.6 h/day**
**Not reaching the WHO recommendations of moderate exercise intensity**	**14.79–30%**
**B. Mental health and stress**	
Depressive symptoms	63.40% (study 1) - 83.3% (study 2) no depression
State anxiety	23.3–41.83%
Perceived stress	mainly stress due to uncertainty
**C.Relationship between mental health, well-being and habitual physical**	
**activity**
Self-reported overall habitual physical activity level (GPAQ) and self-reported depressive symptoms (−)	
Self-reported overall habitual physical activity level (GPAQ) trait as well as state anxiety (−)	
Self-reported overall habitual physical activity level (GPAQ) and body dissatisfaction (−)	
Self-reported overall habitual physical activity level (GPAQ) and self-reported psychosomatic stress symptoms (−)	
Self-reported overall habitual physical activity level (GPAQ) and physical as well as psychological quality of life including aspects of well-being (+)	

*(−): negative correlation, (+): positive correlation. For details see text*.

### Mental Health and Relationship With Physical Activity Among University Students

Importantly, akin to the results obtained for sedentarism and physical activity behavior, the assessment of mental health yielded heightened scores of depression, anxiety and perceived stress among university students. In study 1, only 63.40% of the participants received an average score on the depression inventory [Beck Depression Inventory, BDI-II (65)] indicating no depression. In study 2, 16.7% of the all-female sample reported minimal depressive symptoms (on the BDI-II), while in study 3, depressive symptoms as assessed with the BDI-II ranged on average from 3.85 (no depression)−10.14 (minimal depression). Assessment of trait and state anxiety revealed that in study 1, 29.41% of the participants scored above the clinical cutoff score of trait anxiety and 41.83% reported state anxiety (comparable to clinical samples) and this was confirmed in study 2 in the all-female sample [i.e., 30% (trait) and 23.3% (state) of the participants obtained high scores on trait and state anxiety]. The participants in study 3 reported on average comparable trait anxiety to the participants of study 1 and study 2, respectively. As far as perceived stress is concerned, across studies, perceived stress seemed to be more pronounced in the domains of perceived stress due to uncertainty or excessive demands than perceived stress due to actual negative life events among the study samples of university students.

In accordance with the literature, the results of the RCT studies 1–2 (59) showed significant relationships between mental health and regular physical activity and exercise. Significant negative correlations were found between the participants' self-reported overall habitual physical activity level [GPAQ, (66)] and self-reported depressive symptoms, trait as well as state anxiety, body dissatisfaction and self-reported psychosomatic stress symptoms. Moreover, regular physical activity was positively correlated with well-being and physical as well as psychological quality of life domains. In study 2, relationships between physical fitness and mental health were confirmed by correlations between the participants' cardiovascular fitness [assessed by heart rate variability (HRV) at rest] and the degree of self-reported depressive and anxiety symptoms.

### Health Benefits: Comparison of Exercises Across Studies

When compared across the studies, particularly aerobic exercises (cardio exercise) alleviated depressive symptoms and perceived stress. These effects are seen in women and men when the exercises are carried out regularly, 3 times a week for a duration of 6 weeks at exercise intensities comprising both, low to moderate intensities (study 1). Interestingly, when the same exercise intervention is reduced to 2 weeks and the session number per week are increased (3 sessions per week instead of two sessions per weeks), these effects cannot be found in an all-female sample (study 2). Likewise, the results of study 3 in which a single session of exercise per week was carried out for the period of the winter term and a weekly session comprised a mix of 5–10 min of exercises with low intensity not exclusively focusing on aerobic exercise, this did not significantly change self-reported depression in university students pre to post intervention. In study 2, the exercise intervention comprising balance and motor-coordination exercise only, was not superior to cardio exercise in alleviating perceived psychological stress, depression or anxiety symptoms when carried out for 2 weeks [3 sessions/week, see study 2 in (59)]. However, it improved the participants' performance of motor balance and motor coordination significantly in the all-female sample of study 2 and therefore, not just as known from the literature in the elderly. The participants in study 3 performing low intensity exercise executed for 5–10 min on 1 day per week showed significantly higher overall physical activity and less sedentary behavior at the end of the intervention compared to the participants who engaged in a cognitive intervention of positive writing carried out at the same amount of time and frequency as the exercise intervention (61).

**Figure 5:**
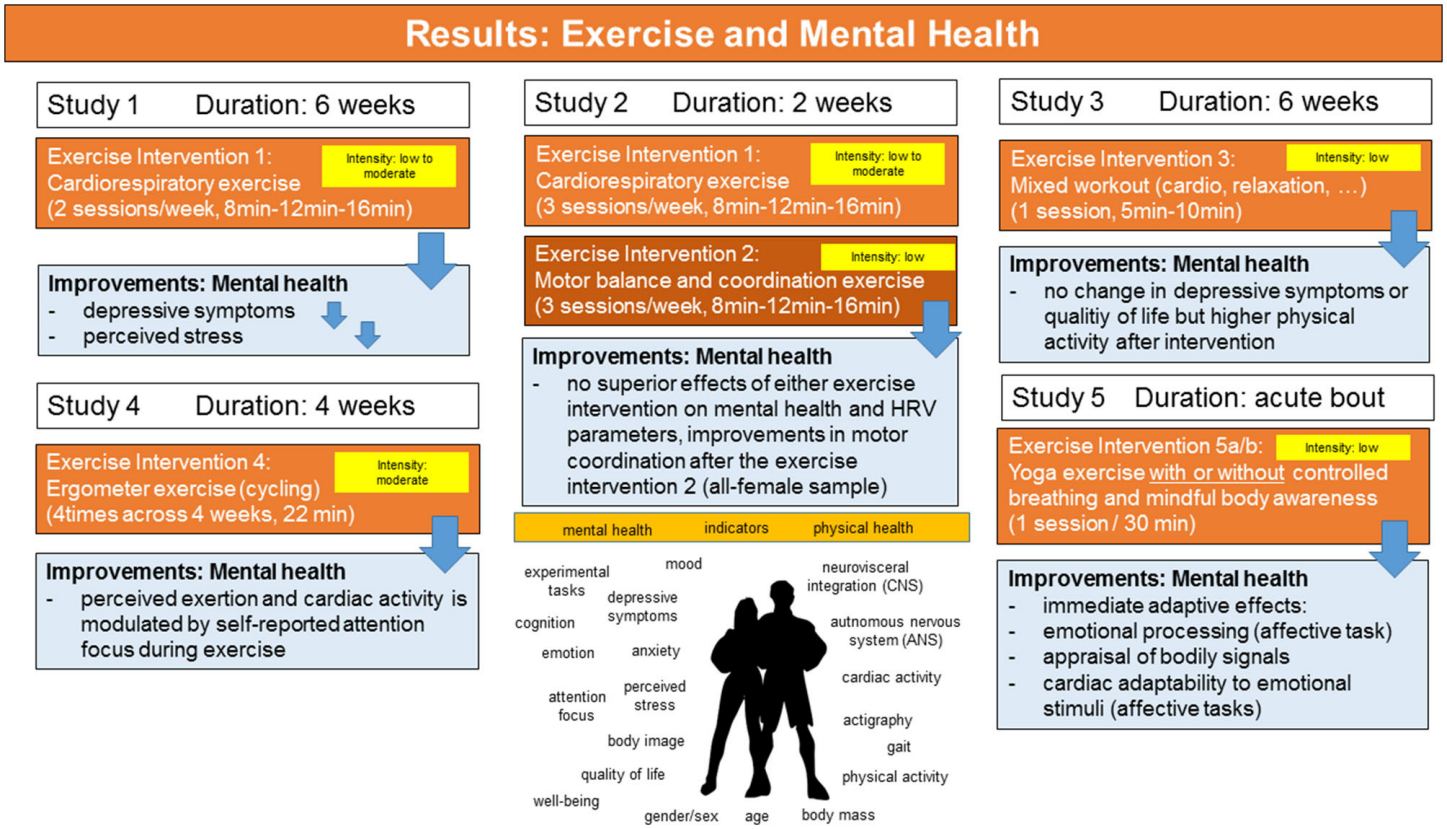
Overview of the results of the exercise interventions on mental health parameters (study samples: university students). For details see text.

Interestingly, four sessions of 22 min of ergometer exercises [cycling at moderate intensity, see (62)] carried out with different attentional focus (internal vs. external) at moderate intensity seem not to have differential effects on cardiovascular activity during the exercise compared to a control condition (no cycling) in a sample of healthy women, however, the self-reported attentional focus during exercise seems to influence both, the degree of exertion from the exercise and the correlations with measures of cardiac activity [see Table 4 in (62)]. In contrast to the cycling exercise used in study 4, the results of study 5 (64) found significant effects on indicators of well-being after a single session of mind-body exercise (yoga). In study 5, women (all university students) performed yoga exercises either with (exercise group 1) or without controlled breathing and mindfulness instructions (exercise group 2) for 30 min. Self-other referential emotional processing, awareness of bodily signals and changes in cardiac activity (heart rate, respiratory sinus arrhythmia, time- and frequency-domain measures of HRV as estimates of parasympathetic cardiac control) were investigated before, during and after the exercise using standardized experimental tasks, standardized questionnaires, and mobile recording devices. This showed that a single session of yoga exercise (of 30 min duration) can facilitate (a) emotional processing, appraisal of bodily signals and cardiac adaptability during the exercise as well as during affective task processing post- to pre-exercise in healthy women. Assessment of cardiac activity showed that mean HR increased during the exercise session. The results of the interventions used in the studies 1–5 are summarized in Figure 5.

## Discussion

There is currently open questions of whether exercise interventions of low to moderate intensity can improve or promote mental health in emerging adults such as university students. This question is the focus of the research project described in this manuscript and its related studies. Emerging adults such as university students are considered vulnerable to mental health conditions, depression and anxiety in particular as well as to the experience of stress [for a discussion, (67)]. Additionally, there is evidence that a majority of emerging adults are not adhering to the physical activity levels recommended in the national and international physical activity guidelines [for metaanalytic results, see (31)]. The results of the already available studies of the research project reviewed above support the hypothesis that university students as emerging adults are suffering from mental health problems including depressive symptoms, anxiety and stress and that among university students, both, the individual physical fitness and the individual regular exercise and physical activity behavior are significantly related to mental health and well-being. Moreover, the studies suggest that these relationships hold true in study samples of university students whose regular physical activity behavior, on average, might range below the weekly physical activity level recommended by the physical activity guidelines. Moreover, the prevalence of sedentary behavior and the amount of habitual physical activity behavior observed in the studies confirm results from previous studies among college students (29) that also support the nationwide trend that a significant majority of the young emerging adult population including university students spends too many hours/days sitting (68). Recent recommendations suggests that sitting more than 8 h/day is as damaging to one's health as obesity or smoking (69).

Of note, all the studies whose results are reviewed in the manuscript were conducted before the COVID-19 pandemic. There is concerns and evidence from research that the mental health of university students might be seriously affected by the consequences of the pandemic. Sadly, this is supported by own survey data (58) collected during the first wave and lockdown in 2020. The results show that compared to the prevalence scores obtained in studies conducted before the pandemic, depression and anxiety as well as threat perception (as type of stress) have increased significantly. Similar results are reported in the surveys conducted in other countries across the world that were affected by lockdowns (34).

The exercise interventions investigated in the different studies summarized in the Results varied in the type of exercises, the frequency and duration of the exercise sessions and the intensity of the exercises. Reviewing the results across the studies allowed giving first answers to the question of when, i.e., at which intensity, frequency and duration low to moderate exercise interventions exert effects on the mental health of university students either immediately after a single exercise session or after repeated and regular exercise sessions. The results of the studies suggest that exercise interventions with a certain frequency (3 times weekly) and frequency and duration of the sessions (6 weeks) and of moderate exercise intensity are best suited to decrease certain mental health problems such as depressive symptoms and perceived stress pre to post intervention in emerging adults (i.e., university students), reporting mild to moderate symptoms in standardized questionnaires and yet not having been diagnosed with a mental disorder [study 1, (59)]. This does however not mean that exercise interventions comprising exercises of low intensity do not have any positive effects on mental health and well-being of university students. Motor balance and coordination training might be effective as well [study 2, (59)]. In addition, even one session comprising a mix of low intensity exercise executed for 5–10 min on 1 day per week might motivate university students to adhere to an active lifestyle with participants taking part in this intervention showing higher overall physical activity and less sedentary behavior at the end of the intervention compared to the participants who engaged in a cognitive intervention of positive writing over the period of a semester [study 3, (61)]. Moreover, the results of study 5 (64) investigating acute bouts of mind-body exercise (yoga) are particularly promising because they support immediate adaptive effects of low intensity exercise (yoga exercise) on both, physical and psychological health parameters. Although effects of additional exercise sessions were not investigated, the results of this experimentally controlled study demonstrate that mind-body exercise of low intensity such as yoga can not only be recommended as an exercise for mental and physical health prevention in the elderly or in certain vulnerable target groups but be recommended among university students as well. The results support the notion that promotion of regular physical activity and exercise of low- to moderate intensity is an important health promotion task in order to help emerging adults such as university students to buffer the psychological, cognitive and emotional demands of pre-dominantly sedentary activities and mental stress.

## Conclusion

### Limitations and Future Outlook

The results briefly summarized in this manuscript await future investigations. In particular, more evidence is needed from studies that use combined examination of psychological and physiological factors to determine the mechanisms of how exercise of low and moderate intensity strengthen mental health and well-being (psychologically and physiologically) and promote an active lifestyle in emerging adults. In light of the so far limited systematic research given (48), the present studies and specifically, the approach of the research project described in this manuscript can be considered a first step into that direction underscoring the importance of the relevance of systematic scientific investigations. Moreover, as proposed by the research project, future studies should focus more on “the individual person” and consider the person's individual preexisting physical activity behavior, the individual's needs, preferences, motivation and reasons for exercise and physical activity behavior. The results and the scientific approach taken by the research project support the notion that theoretical assumptions from exercise psychology, health psychology, but also from neuroscience and emotion and motivation and sports psychology could be helpful when examining mental health effects of exercise interventions. In the long run, the multidimensional approach suggested and approached by the research project can give theoretically guided and personalized exercise and physical activity recommendations for building psychologically-driven exercise interventions that strengthen mental health and well-being of emerging adults in the field of primary prevention. To this end, the investigation of exercise-related effects on mental health should as recommended in this manuscript include a precise description of the exercise and its health benefits along several psychological and exercise dimensions. A detailed and systematic description of both, the exercise itself and its possible health benefits, in addition to a detailed description of the physical activity habits, sedentary behavior, and mental profile of the participants is mandatory for a better understanding of how exercise of different type and intensity influences mental health. Moreover, the purpose of the interventions (e.g., for improving physical health, mental health, or both or other domains such as well-being) should be clearly stated and if relevant may include a description of training principles known from sports psychology and exercise physiology [for an overview see (70, 71)].

Importantly, exercise adherence and dissemination of the interventions also play a role. As outlined in this manuscript, university students as emerging adults display high numbers of activities (job, leisure time) with a high sitting time and sedentary behavior. Therefore, the present research project also includes and provides exercises in a format that can be easily integrated into the daily activity without much need of persuasion or effort. For example the exercises of study 1–3 and study 5 could be carried out as e.g., workout at home or at work. To this end, the individual exercises are videotaped with different exercise models [women and men, e.g., see (59, 60)] at the age of the target groups. Moreover, because guidance of exercise as well as the physical appearance and expertise of the exercise models can have a significant impact on exercise adherence, the individual exercises are also available as standalone (without videos) as digital paper-pencil booklets [see e.g., (60)]. In these exercise manuals, each exercise is given a verbal and a pictorial description of its movements with feedback about its duration, intensity and frequency. The manuals are currently available in two languages, German and English, the latter reducing language and cultural barriers [see e.g., (60)]. The exercises investigated in the research project will be continuously updated, include different types of exercises (as illustrated in Figure 2). Thus, the exercises can be delivered to a broad audience digitally and semi-guided with or without real and symbolic exercise models and with health recommendations from health experts [see (60)].

## Author Contributions

CH: drafting and writing of the manuscript, conceptualization, methodology, validation, formal analysis, resources, writing—review and editing, visualization, project administration, and funding acquisition. The author authored and approved the submitted version of the manuscript.

## Funding

This study was funded by the budgetary resources of the Department of Applied Emotion and Motivation Psychology, Ulm University, Germany.

## Conflict of Interest

The author declares that the research was conducted in the absence of any commercial or financial relationships that could be construed as a potential conflict of interest.

## Publisher's Note

All claims expressed in this article are solely those of the authors and do not necessarily represent those of their affiliated organizations, or those of the publisher, the editors and the reviewers. Any product that may be evaluated in this article, or claim that may be made by its manufacturer, is not guaranteed or endorsed by the publisher.

## Footnotes

^1^Typically, exercises and physical activities with a maximum increase of only 30–39% of the resting heart rate (below 3 MET) are below the physiological threshold at which considerable adaption in performance and physiologic capacity in terms of exercise training effects can be expected. To achieve physical health-related training effects, the physical activities and exercises should preferably include large muscle groups and be above a certain physiological threshold of energy expenditure (EE) >6 MET and 75% of VO_2_ max (maximal aerobic capacity), i.e., the intensity range of moderate exercise.
